# Rapid Root Cause Analysis: Improving OBGYN Resident Exposure to Quality Improvement and Patient Safety Curricula

**DOI:** 10.7759/cureus.56881

**Published:** 2024-03-25

**Authors:** Jessica L Gottula, Erica R Hope, Terra A Wood, Samantha A Medla, Rhiana D Saunders, Erin A Keyser

**Affiliations:** 1 Obstetrics and Gynecology, San Antonio Uniformed Services Health Education Consortium, San Antonio, USA; 2 Gynecologic Surgery and Obstetrics, Brooke Army Medical Center, San Antonio, USA; 3 Gynecologic Surgery and Obstetrics, RAF Lakenheath Hospital, Lakenheath, GBR

**Keywords:** root cause analysis, obgyn residency, graduate medical education (gme), accreditation council graduate medical education, quality improvement and patient safety

## Abstract

Introduction

Each year, millions of patients in the United States experience harm as a result of the healthcare they receive. One mechanism used by health systems to learn how and why errors occur is root cause analysis (RCA). RCA teams develop action plans to create and implement systemic changes in healthcare delivery in order to prevent future harm. The American Council on Graduate Medical Education (ACGME) recognizes the importance of analyzing adverse events, and it requires that all residents participate in real or simulated patient safety activities, such as RCAs. Often, institutional RCAs necessitate the assimilation of participants on short notice and demand considerable time investment, limiting the feasible participation of graduate medical education (GME) trainees. This presents a gap between ACGME expectations and the reality of resident involvement in patient safety activities. We present the first iteration of a quality improvement project encompassing a three-hour resident physician training course with simulated RCA-experiential learning. The purpose of this project was to produce a condensed, educational RCA experience that adequately trains all GME learners to serve as informed healthcare safety advocates while also satisfying ACGME requirements.

Methods

The course (“rapid RCA”) was conducted during protected weekly academic training. All residents of the San Antonio Uniformed Services Health Education Consortium (SAUSHEC) Obstetrics and Gynecology (OBGYN) residency program who had not previously participated in a real or simulated RCA were required to take the “rapid RCA.” Pre- and post-course surveys were completed anonymously to assess baseline knowledge, new knowledge gained from the course, and attitudes toward the course and its importance to resident training.

Results

Fourteen OBGYN residents attended the “rapid RCA,” indicating that 64% (14 out of 22) of the program had no previous experience or opportunity to participate in a real or simulated RCA. Participation in the course demonstrated a significant gain of new knowledge with an increase from 0/14 to 10/14 (71%) residents correctly answering all pre- and post-course questions, respectively (*p < 0.001*). Additionally, on a Likert scale from 1 to 5, with 5 indicating “expert level,” residents indicated they felt more comfortable on patient safety topics after taking the course (mean pre-course score 1.85 to post-course score 3.64, *p < 0.001*). All participants indicated they would prefer to take the “rapid RCA” as opposed to the only available local alternative option for a simulated RCA, currently offered as a full-day intensive course.

Conclusion

A meaningful increase in patient safety knowledge and attitudes toward topics covered in an RCA was demonstrated through the implementation of a “rapid RCA” in OBGYN residents. We plan to incorporate this into our annual curriculum to satisfy ACMGE requirements. This format could be adapted for other specialties as applicable.

## Introduction

Medical errors are made every day in patient care [1]. The exact numbers are difficult to quantify and likely underreported, especially if they do not result in patient harm. A review and meta-analysis in 2019 estimated that one in 20 patients is exposed to preventable harm in medical care [2]. However, not all errors cause patient harm. Some errors, called “near misses,” still represent errors but are caught and corrected prior to any harm reaching the patient. The National Patient Safety Foundation (NPSF) published recommended practices to improve learning from adverse events, errors, and near misses to prevent future occurrences [3]. One mechanism used by healthcare systems to achieve this is root cause analysis (RCA) [4,5]. RCAs bring together a multidisciplinary team to identify process failures or system errors following an adverse event [6]. The team reviews data, interviews involved personnel, identifies root causal factors, and ultimately develops corrective actions for each causal factor. The RCA review is non-punitive, and systemic action plans are focused on preventing future harm.

The Accreditation Council for Graduate Medical Education (ACGME) has implemented “milestones” to assess resident competency of pre-determined educational objectives [7]. One of the systems-based practice requirements involves “conducting an analysis of patient safety events and offering error prevention strategies (simulated or actual).” Participation in patient safety activities is also stressed in the annual resident and faculty ACGME surveys. Often, institutional RCAs necessitate participant recruitment on short notice and demand considerable time investment, limiting the feasible participation of graduate medical learners, especially in surgical specialties [8-11]. In an effort to increase graduate medical education (GME) involvement and exposure to RCA, our institution facilitates a large-scale, full-day RCA course that facilitates a simulated RCA using historically examined and closed cases that occurred within our institution. Participation in the course requires approximately five hours of pre-course work followed by undisturbed participation in a full-day simulated RCA with small-group RCA team activities. This event includes interviewing actors and creating corrective action plans. However, given the rigors of residency training, trainee involvement can be very limited, with only 26% (68 residents of 261) of graduating residents being able to participate in this simulated RCA course and only 6% (four of 68 participants) of those represented by surgical specialties. Thus, in an effort to address this gap between ACGME expectations and the reality of resident involvement in patient safety activities, we constructed a three-hour resident physician training course termed “rapid RCA.” We present the first iteration of the quality improvement project with simulated RCA-experiential learning. The purpose of this project was to produce a condensed, educational RCA experience that adequately trains all our GME learners to serve as informed healthcare safety advocates while also satisfying ACGME requirements. Our specific aim was for our program to achieve 100% resident participation in an RCA-simulated course in the academic year 2022-2023.

## Materials and methods

We constructed a three-hour “rapid RCA” that teaches key analytical and teamwork skills through a combination of didactic and interactive small-group exercises that simulate an RCA investigation of a historical obstetric case. The purpose of this project was to create a condensed RCA course on a timescale that is feasible for resident participation and that both appropriately exposes residents to patient safety event analyses and satisfies ACGME graduation requirements. Our outcome measures included total resident course participation across the program, pre- and post-experience knowledge assessments, and resident perception of the course’s educational value. Our process measures included time spent to develop the curriculum, the number of personnel needed to facilitate the course, and the resources required to complete the training. Balancing measures comprised the required protected time away from clinical duties or other academic activities in order to conduct the course. 

The course was created by a multidisciplinary team including five obstetrics and gynecology (OBGYN) physicians and a labor and delivery nurse with consultation from the institutional assistant dean of quality improvement patient safety and the department quality assurance and process improvement coordinator who provided RCA subject matter expertise [12,13]. A historical case from the OBGYN department was selected for its relevance to the learner; deidentified medical records from the incident case were pared down for presentation in this shortened format. Additionally, we created interview scripts for important key players in the case (e.g., emergency room intern, attending OBGYN, labor and delivery nurse, and pharmacist) that our learners could use during the simulated RCA for gathering additional information pertinent to the event. This interactive “rapid RCA” was designed to introduce foundational concepts that will allow learners to identify and implement sustainable system-based improvements with the goal of enhancing patient safety across diverse settings and along the continuum of care [8]. As part of the development process, a program implementation guide, participant worksheets, and facilitator guide were created and are presented in Appendix 1.

Table 1 describes the important steps of an RCA that we aimed to simulate with this curriculum and the timeline followed to keep the “rapid RCA” at three hours.

**Table 1:** Rapid root cause analysis course outline

Rapid root cause analysis step	Activity description	Time allotted
Root cause analysis introduction	Pre-course survey followed by facilitator-led didactics on basics of patient safety events and root cause analysis	10 minutes
Case introduction	Brief summary of patient case	5 minutes
Group exercise 1: medical records review and timeline creation	Collect facts surrounding case using electronic medical records and identify processes that possibly led to event	30 minutes
Conduct interviews	Additional information requested from key players in case	20 minutes
Group exercise 2: identify causal factors	Create causal factor statements with contributing factors that led to an effect which increased the likelihood of the event	35 minutes
Group exercise 3: develop corrective action plans	Identify ways to redesign the system processes that prevent this event from occurring again that include verification metrics, milestones and points of contact	20 minutes
Conclusion	Discussion and post-course survey	15 minutes

At the start of the course, a 10-minute didactic lesson was given to orient the residents to the basic steps of the RCA process. To accurately simulate a real-world RCA, the residents were provided with deidentified medical records. In groups of three, using the medical records, they identified potential critical points in the case of patient harm and practiced creating a process map visual timeline. Additional information was available if requested by the group in the form of interviews with specific team members involved in the case. Residents then practiced developing causal factors that help explain why an event occurred, connecting cause with effect. Lastly, the groups were asked to brainstorm corrective action plans targeted at each of the causal factors identified, including measuring the success of changes implemented.

This study was conducted at Brooke Army Medical Center, San Antonio, TX, during the 2022-2023 academic year, and it targeted the San Antonio Uniformed Services Health Education Consortium (SAUSHEC) OBGYN residency program. The course was conducted during protected, weekly educational training. All residents in the OBGYN program who had not previously participated in a real or simulated RCA were required to take the “rapid RCA.” The course was facilitated by the working group who designed the program, including both subject matter experts. Anonymous pre- and post-course surveys were completed to assess baseline knowledge, new knowledge gained from the course, and attitudes toward the course and its perceived importance to resident training. Results were collected and analyzed, and comparative statistics were used to assess the difference between pre- and post-course knowledge and attitudes. For non-parametric data, the Wilcoxon signed-rank test was used to compare pre- and post-survey results. A p-value of <0.05 was considered significant. The statistical software package JMP©, version 16 (SAS Institute Inc., Cary, NC, 1989-2023) was used for all data analysis.

## Results

**Figure 1:**
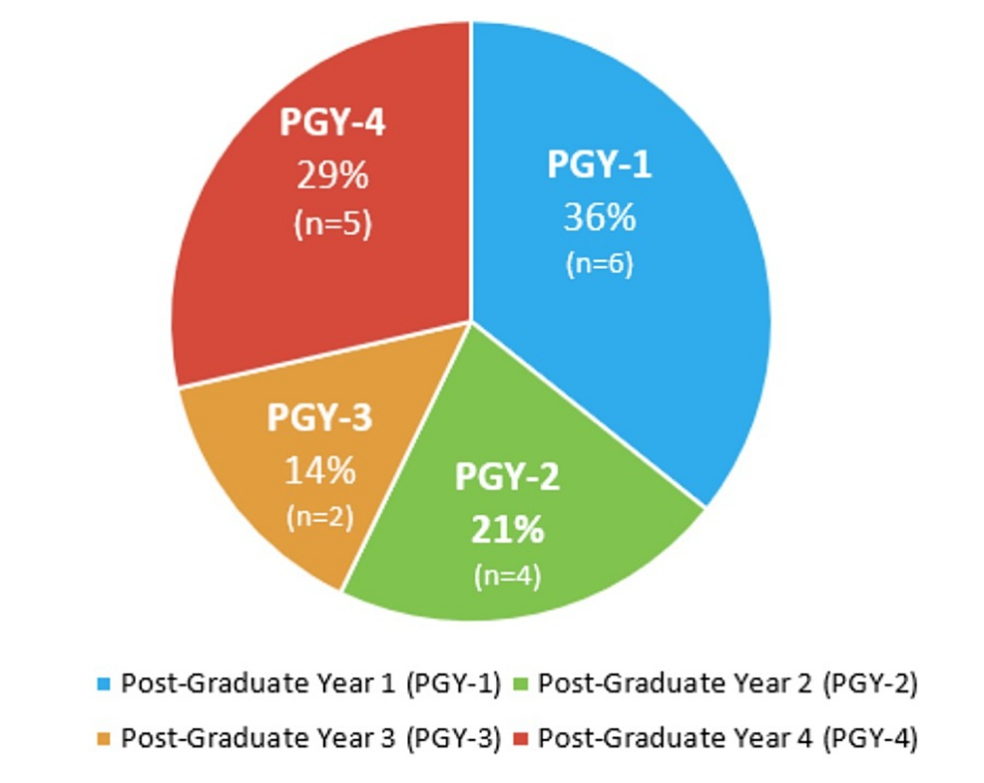
Resident participation in “rapid root cause analysis” by class year (n = 14)

Fourteen OBGYN residents attended the “rapid RCA,” representing 64% of the program, who had not yet had the opportunity to participate in a real or simulated RCA despite being an ACGME requirement. Figure 1 depicts the resident year-levels that participated with the post-graduate year (PGY)-1s making up 36% (6/17) of participants, PGY-2s representing 21% (4/17), PGY-3s at 14% (2/17), and PGY-4s encompassing the remaining 29% (5/17).

**Figure 2:**
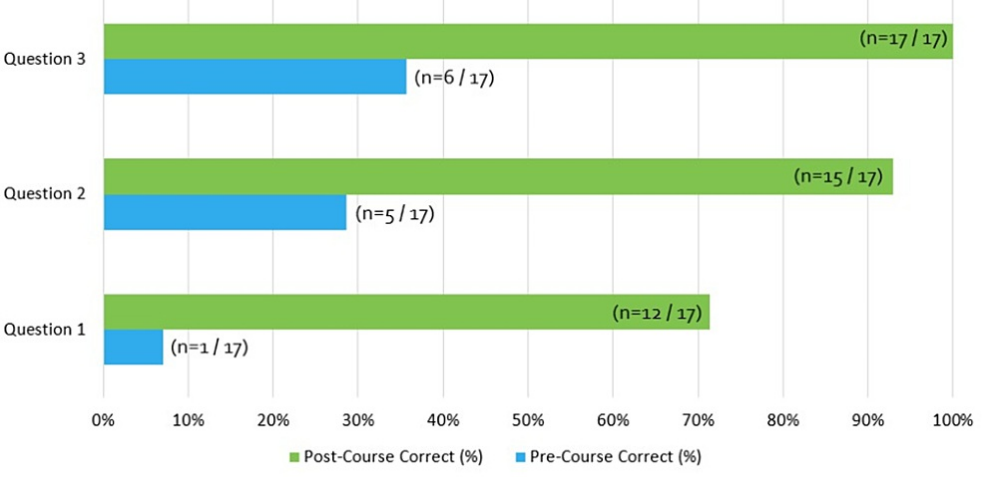
Pre- and post-rapid root cause analysis knowledge assessment performance

The knowledge-based quiz assessing information presented in the “rapid RCA” was given to the residents before and after the presentation. Involvement in the course demonstrated a significant gain of new knowledge with an increase from 0/14 to 10/14 (71%) residents correctly answering all questions, respectively (p < 0.001), as shown in Figure 2.

**Figure 3:**
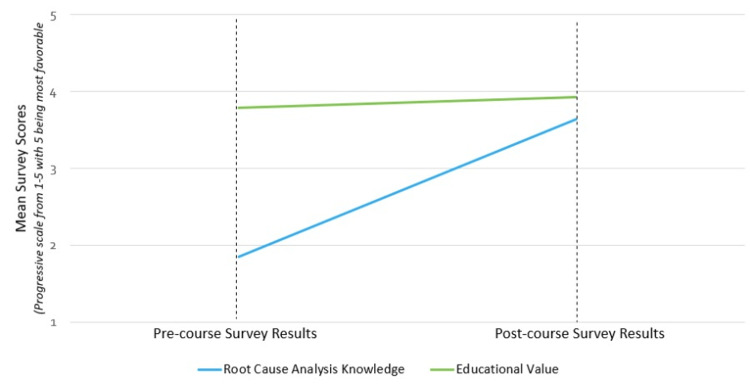
Pre- and post-course knowledge perception and anticipated educational value results

Additionally, on a Likert scale from 1 to 5 (5 indicating expert level), residents indicated they felt more comfortable on patient safety topics after taking the course (mean pre-course score 1.85 to post-course 3.64, p < 0.001), as illustrated in Figure 3. Pre- and post-course, all participants rated the “rapid RCA” very valuable on a scale from 1 to 5 (5 indicating highly valuable), with an average pre-course score of 3.79 compared to 3.93 post-course, as shown in Figure 3.

While this is not a statistically significant difference, it does illustrate the perceived importance of learning about patient safety and quality improvement by the learners. All participants indicated they would prefer to take the “rapid RCA” as opposed to the only available local alternative option for a simulated RCA, currently offered as a full-day, intensive course with required pre-course work. The “rapid RCA” was completed in February 2023, just prior to the distribution of the annual ACGME resident survey. The survey question that involves “participation in safety event investigation and analysis” increased from 76% (16/22) in 2022 to 100% (22/22) in 2023, validating our results.

## Discussion

This department-specific “rapid RCA” delivered during protected educational time increased compliance with the ACGME requirements that all graduating residents participate in a real or simulated RCA from 36% (eight of 22 residents) to 100% in a military OBGYN residency program. While it was expected that the greatest representations of PGY class who had not yet met this ACMGE requirement were interns (36% of course attendees, 6/17), it was very unexpected that the next highest group of attendees were PGY-4s at 29% (five of 17 attendees). This highlights the gap between what is considered an integral part of training as mandated by the ACGME and the reality of our trainees, especially in a surgical specialty of having the opportunity to meet this requirement [14]. 

Meeting the graduation requirement is important, but arguably more important is ensuring the fundamental concepts of patient safety and RCA. This course demonstrated that residents’ understanding of fundamental patient safety and RCA concepts was suboptimal prior to taking the course, with zero residents being able to answer all knowledge questions correctly on the pre-course survey. Demonstration of knowledge on these topics was significantly increased when comparing pre- and post-course surveys, increasing to 71.4% (12 of 17 attendees) on the post-assessment being able to answer all questions correctly. RCAs are an integral part of the healthcare landscape today, and they are required at the hospital level for review of all sentinel events as determined by the Joint Commission [15]. Understanding these concepts and the ability to incorporate them into practice is an integral part of residency training, particularly in surgical specialties. While morbidity and mortality conferences (MMC) have long been a tool used in surgical subspecialties to review adverse events or undesired outcomes, they often lack the infrastructure to evaluate system vulnerabilities outside the purview of the individual surgeon or service that contributes to the outcome. RCAs are designed with that exact goal in mind and thus are optimally positioned to enhance surgical education in addition to MMC, concepts of quality assurance, and teaching of patient safety skills in a surgical residency [16]. Not only was this course successful in teaching the basic principles of an RCA, but residents also expressed that they felt more comfortable employing these concepts in their own practice post-survey, proving the educational value of this course.

A major strength of our study is the feasibility, practicality, and reproducibility of this curriculum. This is a ready-made, specialty-relevant curriculum that not only teaches an important aspect of medicine but also meets the ACGME requirements for graduation in a meaningful way. Additionally, a strength is the format in which the course was developed, utilizing subject matter experts in patient safety to ensure reliable content, case selection relevant to the target audience, and involving residents in the creation of the curriculum as ultimately it is designed for them. Lastly, we formatted the content in such a way that it can be easily adapted and implemented in other OBGYN residency training programs as well as in other specialty training programs.

We recognize that the limited external validity of this project is a limitation of the study. However, as mentioned, this packaged and ready-made curriculum can be easily modified and tailored to meet the needs of another GME program, as provided in Appendix 1. In fact, this curriculum was adapted to emergency medicine within our institution and in other OBGYN programs outside of our institution. Limited data from implementation at another location resulted in similar results as our program, with a demonstration of increased knowledge both subjectively and objectively, as well as an increased appreciation for the value of the RCA content by residents taking the course. Finally, the knowledge assessment from our RCA project represents a single snapshot obtained immediately after the first iterative course completion. For true retention of information, follow-up assessments are encouraged.

## Conclusions

Implementation of a department-level “rapid RCA” was feasible and effective for meeting ACGME requirements and resulted in a meaningful increase in patient safety knowledge and attitudes toward RCA. This course has been adapted to other specialties in GME at our institution and beyond, demonstrating the potential to extend an impactful patient safety curriculum across all fields.

## Appendices

Appendix 1: the entire rapid-RCA curriculum can be accessed at https://drive.google.com/drive/folders/1nm-z9xegiAa14ZQrDIjfVBoH4PPVlB13 

The authors encourage all programs to utilize the resources provided and welcome any questions or concerns regarding the implementation of this course.
